# New Platforms Based on Frontal Cellular Automata and Lattice Boltzmann Method for Modeling the Forming and Additive Manufacturing

**DOI:** 10.3390/ma15217844

**Published:** 2022-11-07

**Authors:** Łukasz Łach, Dmytro Svyetlichnyy

**Affiliations:** AGH University of Science and Technology, Faculty of Metals Engineering and Industrial Computer Science, al. Mickiewicza 30, 30-059 Krakow, Poland

**Keywords:** recrystallization, phase transformation, rolling, additive manufacturing technologies, frontal cellular automata, lattice Boltzmann method

## Abstract

Materials science gives theoretical and practical tools, while new modeling methods and platforms provide rapid and efficient development, improvement, and optimization of old and new technologies. Recently, impressive progress has been made in the development of computer software and systems. The frontal cellular automata (FCA), lattice Boltzmann method (LBM), and modeling platforms based on them are considered in the paper. The paper presents basic information on these methods and their application for modeling phenomena and processes in materials science. Recrystallization, crystallization, phase transformation, processes such as flat and shape rolling, additive manufacturing technologies (Selective Laser Sintering (SLS)/ Selective Laser Melting (SLM)), and others are examples of comprehensive and effective modeling by the developed systems. Selected modeling results are also presented.

## 1. Introduction

Materials science and engineering are focused on creating new substances or changing the physical and chemical composition of existing materials to improve their properties. The important manufacturing techniques of product manufacturing are forming and additive manufacturing. Forming processes are important for the production of lightweight components made of metallic materials with defined geometric parameters and mechanical properties. Additive technologies have been introduced in many facilities in the preparation of short-term production (parts with complex geometry), and their use allows for saving materials, time, and production costs of various components. The preparation of forming processes and additive manufacturing using a trial-and-error method is a thing of the past. Today, methods that include computer-aided design systems (CAD) are more effective. The application of different numerical methods for the simulation of different phenomena in materials has become incredibly important in the last few years. The first method that is often used for such simulations is the phase field (PF) method. PF was recently used, for example, for the morphological and microstructural evolution of metallic materials under environmental attack [1]. DeWitt and Thornton presented a brief introduction to phase-field modeling and possible applications for simulations of precipitate evolution, grain growth, solidification, phase separation in battery electrodes, and deposition [2]. Recent applications of phase-field simulations of solid-state microstructure evolution and solidification that have been compared and/or validated with experiments were described by Tourret et al. [3]. The level-set (LS) method can be used, for example, to simulate grain growth with an evolving population of second phase particles [4] and for modeling multiphase thermo-fluid flow in additive manufacturing processes [5]. A review of level-set methods and some recent applications can be found elsewhere [6]. The Monte Carlo (MC)–Potts model was used to model the primary recrystallization and grain growth in cold-rolled single-phase Al alloy [7] and grain growth simulation of single-phase systems [8]. The finite element method (FEM) was recently used for the analysis of microstructure evolution and mechanical properties during compression of open-cell Ni-foams with hollow struts [9], for simulations of microstructure evolution in single crystal and polycrystal shape memory alloys under uniaxial tension and compression [10], and for predicting phase transformations and microhardness for directed energy deposition of Ti6Al4V [11]. The cellular automata (CA) [12,13,14] method can be used, for example, for simulation of dynamic recrystallization behavior under hot isothermal compressions for as-extruded 3Cr20Ni10W2 heat-resistant alloy [12], modeling of solidification microstructure evolution in laser powder bed fusion-fabricated 316L stainless steel [13], simulation of coupled hydrogen porosity, and microstructure during solidification of ternary aluminum alloys [14]. Other examples of used numerical methods to model the evolution of microstructure in materials are analysis of metal extrusion by the finite volume method (FVM) [15], prediction of multidirectional forging microstructure evolution of GH4169 superalloy by the neural network [16], and prediction of microstructure evolution with convolutional recurrent neural networks [17]. Multiscale models, which are a combination of several methods (for example, finite element and cellular automata or cellular automata and finite volume), are also used. FE and CA methods were recently used for numerical prediction of microstructure for selective electron beam melting [18], while CA and FVM were used to predict the grain structure of an alloy, e.g., Inconel 718, fabricated by additive manufacturing [19].

Considering the use of a given method to model a given phenomenon or process, its advantages and disadvantages are always important. The phase-field method automatically takes into account changes in front topology and generalizes easily to 3D areas; however, there is a complicated theoretical side of the model, small model volumes, and the need for fine discretization in the front area. The level-set method allows for a direct representation of the interface and curvature of the grain boundary, and there is also no need to discretize explicitly the interface. LS has an inability to track the evolution of the texture, which can be solved by combining LS with the theory of crystal plasticity. The numerical implementation of the Monte Carlo algorithm is quite simple, and good computational performance can be achieved using parallel calculations; however, the MC solutions depend on the type of mesh used, a good representation of the curvature of the grain boundary, the proper determination of the simulation length, and the time scale. Taking into account the finite element method, it can be seen that complex shapes can be approximated with high accuracy, the sizes of the elements can be different, and nonlinear boundary conditions can be defined. FEM needs to control the numerical error because it depends on discretization parameters, material properties, and boundary conditions. CAs provide very high computing performance and a better accounting of many factors, including local, e.g., crystallographic orientation, or dislocation density. The simple rules and synchronous updating of cell states on the grid introduce additional simplifications in the modeled reality, and the calculation time is the main preference for the use of 2D solutions compared to 3D.

Recently, one of the most effective methods for simulations of different phenomena and processes in materials science is the frontal cellular automata (FCA) and the lattice Boltzmann method (LBM). FCA is a modified CA algorithm that allows for algorithmic acceleration of calculations with the same level of accuracy. The calculation time of the 3D FCA calculations is similar to the time of classical 2D CA. LBM is very feasible for modeling the flow of liquids, gases, mixtures, heat flow, and transfer as well as diffusion occurring simultaneously with the liquid (gas) flow. The wide application of the LBM method was limited by some of its features. The main disadvantages were the high memory volume requirements and a large number of iterations, i.e., the computation time even in two-dimensional and especially in three-dimensional calculations. The development of computer and computational technology related to the possibility of using parallel computing with the use of graphics cards has changed the perception of the LBM method from being a little useful to very effective. LBM was recently used, for example, for modeling the evolution of coherent vortices and periodic flow in a continuous casting mold [20], simulating fluid flow in multi-scale porous media [21], and simulating fluid flow in multi-scale porous media [22]. LBM comes from the CA, so a combination of these two methods in comprehensive solutions can be considered natural. Taking into account their algorithmic structure, these methods are almost ideal for a parallel programming environment involving CUDA (Compute Unified Device Architecture) graphics cards. Models based on LBM and FCA do not need a complicated interface. The solution can be implemented in a common domain, while the number of processes and phenomena modeled simultaneously depends on the number of variables associated with a point, cell, or node in the same model volume. Extending the model with a new process or phenomenon consists of adding variables and an algorithm without developing additional communication between different parts of the model. This is an undeniable advantage compared to multiscale models. FCA and LBM can be the base methods for the development of comprehensive platforms to model various technological processes.

A frontal cellular automata (FCA) algorithm was developed by Svyetlichnyy and for the first time was successfully applied to model microstructure evolution in 2010 [23]. Over the next years, the method has been successfully used to model different phenomena and processes in materials science. Crystallization, recrystallization, phase transformations, severe plastic deformation, and technological processes such as rolling or continuous casting were successfully modeled. Universal frontal cellular automata can be used for practically all possible processes consisting of nucleation and grain growth. Closed-transition circuits make the FCA suitable for modeling multiple nucleation and grain growth.

The introduction of the lattice Boltzmann equation to lattice gas automata (LGA) applied to viscosity calculations can be regarded as the first of the milestone steps leading to the LBM method. The next steps were the replacement of particles by the continuous Fermi–Dirac distribution for the equilibrium distribution, linearization of the collision operator, application of the Boltzmann distribution instead of the Fermi–Dirac distribution, and replacement of the collision operator with the BGK (Bhatnagar–Gross–Krook) approximation. After 1992, the LBM method can be considered as developed. Its further development is primarily related to the expansion of application areas and the development of new principles, methods, etc. Materials science and engineering are now important areas of FCA and LBM application, and further dynamic growth of interest in these methods is expected soon.

The paper presents the new modeling platforms that allow for the simulation of different phenomena and processes in materials science as well as forming processes (for example, rolling processes) and additive manufacturing technology. Basic information on the FCA and LBM methods, which are the basis of these platforms, is shown. The principles of calculations carried out with the use of these methods are presented. Finally, examples of modeling results of phenomena within the considered processes and the results of simulation of industrial processes in the general aspect obtained by using the developed platforms are shown. The presented computing platforms are based on their software developed based on FCA and LBM methods. Calculations can be performed on Windows 8, 10, and 11 and Linux operating systems. Some of the new algorithms developed include the possibility of parallel computing with the use of CUDA (Compute Unified Device Architecture), i.e., the universal architecture of multicore processors on Nvidia cards and the programming environment based on the C and C++ programming language, which is an integral part of this architecture. Some of the developed software is also a CPU-based sequential computing version, written in C++ or Fortran. For parallel calculations, graphics cards (GeForce RTX 2080 Ti, GeForce TITAN 6GB, TITAN Z 12 GB, GeForce 1080Ti, GeForce 1060, NVIDIA, Santa Clara, California, USA) are used. Processing and presentation of the results are carried out primarily with the use of OpenGL technology.

## 2. Frontal Cellular Automata

Frontal cellular automata (FCA) are a modernization of the classical cellular automata and their development began in the 2000s. FCA is very efficient The method is very effective for a certain class of tasks, in which changes can be presented and described as the movement of boundaries or the movement of the front of changes, while the rest, a significantly larger part of the space, remains unchanged. This principle can be applied, for example, to modeling the evolution of microstructure. The principle of considering only the front of changes gave the name to the modified method.

**Figure 1:**
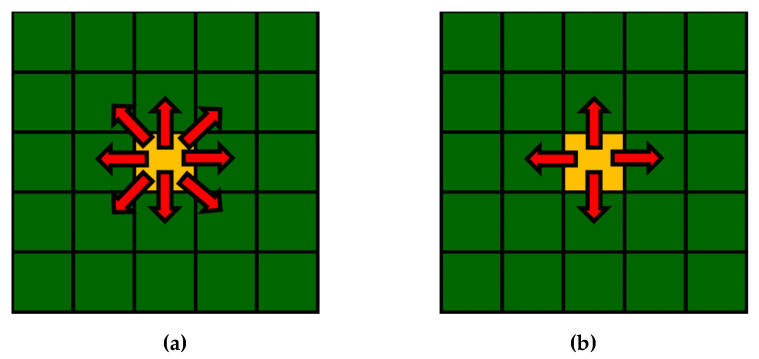
FCA communication direction using the Moore (**a**) and von Neumann (**b**) neighborhoods.

There are several differences between the classical CA and the FCA. Three of the most important modifications are increasing the number of states (multiple states), inverse information transfer, and using linked lists. The increasing number of states differentiates the processing procedures of cells in different states. The inverted transfer allows sending only critical information at an appropriate moment to the neighboring cell that influences its states rather than collecting mainly unused information from the whole neighborhood in each iteration. Figure 1 presents the direction of information transfer considering the Moore and von Neumann neighborhoods. The linked lists allow cells to proceed only in “active” states on the front of changes. These and other means exclude a huge number of cells from calculations in every iteration, reducing overall computation time by hundreds and even thousands of times.

**Figure 2:**
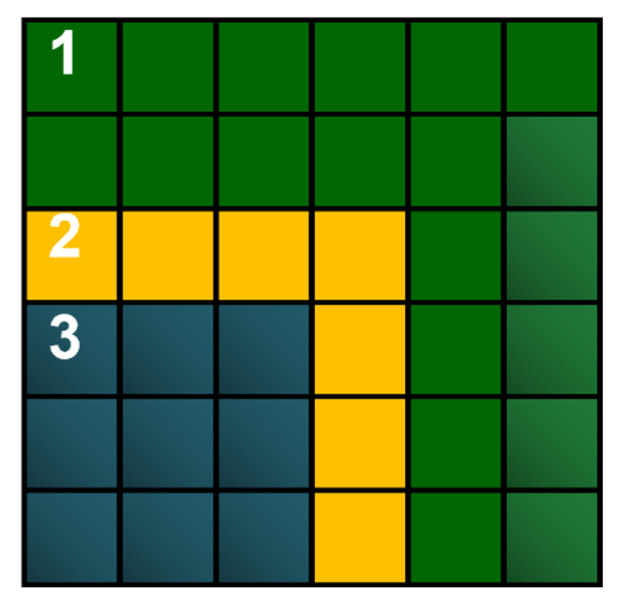
The front of the growing grain with cells in the state: 1, initial; 2,frontal (transitional); 3, final.

Figure 2 shows a fragment of the space with cells in three states. The initial state of the cell does not change if there are no changes in its neighborhood and changes if a cell in the frontal state appears in its neighborhood. The cell in the frontal state changes its state in the final state immediately or with delay, depending on a modeling phenomenon or process. The final state never changes. As a result, only frontal cells are used for calculations. The introduction of the frontal state allowed us to resign from exploring the entire space and to concentrate activities on cells in which changes take place.

The described modifications reduce the computation time but increase the complexity of the algorithm. FCA creates, among other things, the possibility to take into account changes in shape and sizes of the cells, reorganize space, and introduce new structures that connect cells in the group of the same state, in the same grain, or according to other arbitrary features. In FCA, linked lists usually unite cells in the same state by adding and removing elements. It is a central point in the acceleration of calculation, especially for sequence programming. Reducing the calculation time of the frontal automata compared with the classical one is significant in the 2D models, and it is radical in the 3D models. More detailed information on FCA can be found elsewhere [23]. However, it should be noted that the advantage of FCA over classic CA is slightly smaller for parallel computations because there are no effective methods of linked-list maintenance.

## 3. Lattice Boltzmann Method

The lattice Boltzmann method (LBM) is very feasible for modeling the flow of liquids, gases, mixtures, heat flow, and transfer as well as diffusion occurring simultaneously with the liquid (gas) flow. LBM is derived from a variant of the CA method, which can be regarded as a simplified model of molecular dynamics that implements the discretization of space, time, particle velocity, and the statistical Boltzmann equation. The basis of the method is the solution of the transport Boltzmann equation:

(1) $$\frac{\partial f}{\partial t}+\frac{\partial f}{\partial x}\cdot \xi +\frac{F}{m}\cdot \frac{\partial f}{\partial u}=\Omega$$

where *f*—particle distribution function; ***x*** and ***ξ***—phase space variables (coordinate and velocities); *t*—time; ***F***—external force (for example gravity); *m*—mass; ***u***—macroscopic velocity; *Ω*—the collision operator.

The particle distribution function along the directions and velocities is a Maxwell function, so along any direction of space, particles have velocities according to the Gaussian distribution:

(2) $$f=\frac{\rho }{{\left(2\pi RT\right)}^{D/2}}exp\left(-\frac{{\left(\xi -u\right)}^{2}}{2RT}\right)$$

where ***ξ***—microscopic velocities; *R*—gas constant; *D*—space dimension; *ρ*—gas density; *T*—temperature.

The particles velocities space ***ξ*** can be reduced to a finite (discrete) velocity system {***e**_i_*, *i* = 1,…,*b*}, and a grid can be built on which calculations can be made. Therefore, the equation is approximated for the characteristic velocities of the determined grid and the selected directions:

(3) $$f_{i}\left(x+e_{i},t+1\right)=f_{i}\left(x,t\right)+\Omega_{i}$$

Equation (3) is called the lattice Boltzmann equation (LBE) and is the basis of the LBM method. An important element of this equation is the collision operator. One of the first and possibly the simplest and most frequently used is the operator proposed by Bhatnagar, Gross, and Krook (named after BGK), which has the following form:

(4) $$\Omega_{i}\left(f\right)=\frac{\Delta t}{\tau }\left[f_{i}\left(x,t\right)-f_{i}^{eq}\left(x,t\right)\right]$$

where Δ*t* = 1—time step; *τ*—relaxation time; *f^eq^*—the distribution function in the appropriate direction in the equilibrium state.

The solution of Equation (3) with the collision operator (4) is divided into two stages: collision and advection (streaming). Calculations for the collision stage are preceded by the calculation of macroscopic variables: density *ρ* and velocity ***u*** (through momentum *ρ**u***):

(5) $$\rho =\overset{b}{\underset{i=0}{\sum }}f_{i}$$

(6) $$\rho u=\overset{b}{\underset{i=0}{\sum }}f_{i}e_{i}$$

and the equilibrium distribution *f^eq^*:

(7) $$f_{i}^{eq}\left(x,t\right)=w_{i}\rho \left(1+\frac{u\cdot e_{i}}{c_{s}^{2}}+\frac{{\left(u\cdot e_{i}\right)}^{2}}{2c_{s}^{4}}-+\frac{u\cdot u}{2c_{s}^{2}}\right)$$

where *c_s_*—sound speed in the modeled flow; *w_i_*—directional weights.

The output distribution function after the collision has the following form:

(8) $$f_{i}^{out}\left(x,t\right)=f_{i}^{in}\left(x,t\right)-\frac{1}{\tau }\left[f_{i}^{in}\left(x,t\right)-f_{i}^{eq}\left(x,t\right)\right]+F_{i}$$

On the other hand, at the advection stage, a simple transfer of the distribution function to neighboring nodes takes place:

(9) $$f_{i}^{in}\left(x+e_{i},t+1\right)=f_{i}^{out}\left(x,t\right)$$

An additional stage of the calculations is considering the boundary conditions.

**Figure 3:**
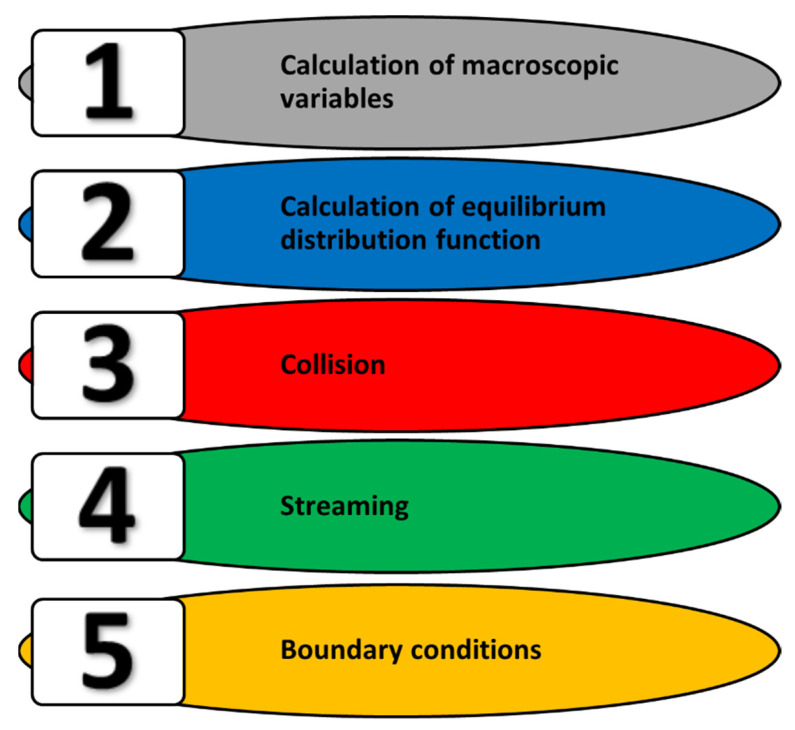
Structure of the LBM calculation algorithm.

The same LBM algorithm is used for all issues, including cyclically repeating steps (Figure 3):

1. Calculation of macroscopic values: density *ρ*, velocity *u*, temperature *T*, concentration *C*, etc.;
2. Determination of the equilibrium distribution function *f^eq^* for the modeled variable;
3. Collision operation, determination of the output distribution function *f^out^*;
4. Streaming operation, transfer of the distribution function to the appropriate cells, site, or nodes;
5. Consideration of boundary conditions.

Some of these stages can be combined (for example, 1 + 2 + 3 or 4 + 5). The cycle can be started from anywhere although it starts with the determination of macroscopic values, the determination of equilibrium distributions functions, and the assignment of output functions not according to the collision operator but with the equal equilibrium distribution *f^out^* = *f^eq^*. In this way, the computation starts from the local equilibrium state in all nodes, and changes occur due to the global imbalance.

The basis of the method is the discretization of space and time. A regular square (two-dimensional) or cubic (three-dimensional) grid with a distance between adjacent nodes equal to one is superimposed on the space. The length of the time step is equal to one. An important element of the system is the selection of the velocity model. In one-dimensional space, the models D1Q2 and D1Q3 are used; in a two-dimensional space, D2Q4, D2Q5, and D2Q9 are used. For three-dimensional problems. D3Q6, D3Q7, D3Q15, D3Q19, and others are used. D indicates the dimensionality, while Q indicates the number of velocities.

More information on the LBM method can be found elsewhere [24].

## 4. Platform Based on Frontal Cellular Automata for Modeling the Microstructural Phenomena in Technological Processes

The use of any method, including cellular automata, to model a specific technological process requires formalization and solving several problems directly related to the limitations of its applicability. The accurate analysis allows for adequate and effective use of a given modeling method. By determining and accurately describing selected issues, microstructural phenomena, and processes, it is possible to develop an appropriate scheme of numerical modeling.

**Figure 4:**
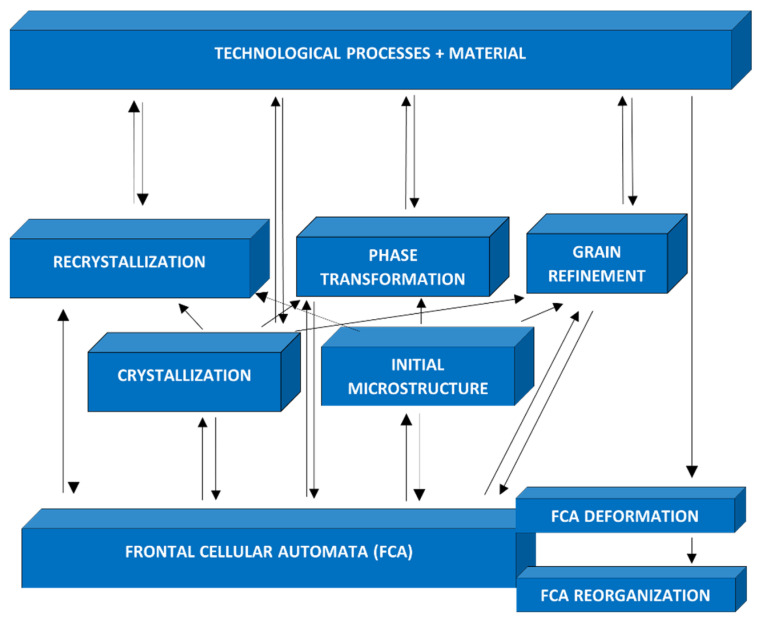
Structure of the platform for modeling microstructure evolution in technological processes.

Choosing as a goal to create a universal tool for modeling microstructure evolution in various technological processes, e.g., casting, forming, heat treatment, etc., a platform based on frontal cellular automata was developed (Figure 4). This FCA platform was created as a hierarchical system and used for the study of microstructural phenomena to design, improve, and optimization of technological processes, including crystallization, recrystallization, grain growth, and phase transformation. The basis of the platform (first level) is frontal cellular automata (FCA) augmented with the transformation and reorganization of cells. The models creating the second level and responsible for the simulation of basic microstructural phenomena are based on FCA. The third level of the system includes models of technological processes and uses the models of the second level as building blocks. An additional element that complements the system is the material database.

The development of the platform included several stages according to its hierarchical structure, from the development of FCA algorithms and models of individual phenomena to the final comprehensive model of the technological process. In the beginning, the second-level models (Figure 4) were simplified, schematically reflecting real phenomena. Then, all elements of the platform were constantly expanded after a wide range of experimental and theoretical research. Models of the second level can be divided into two groups. The models of the first group create the original structure. They are the model of the «real» crystallization phenomenon (solidification) [25] and the «unreal» process of creating the initial microstructure. Both models do not require the initial microstructure, and the results of their simulation, i.e., the initial microstructure, can be used by the models of the second group. Models in the second group require initial microstructure, and the group includes models of recrystallization, phase transformations, and microstructure refinement. The phenomena modeled on the second level can be divided into two stages: nucleation and grain growth (crystals, crystallites, and a new phase), so they can be realized using the same FCA (considering the relevant details).

### 4.1. FCA Deformation and Reorganization

**Figure 5:**
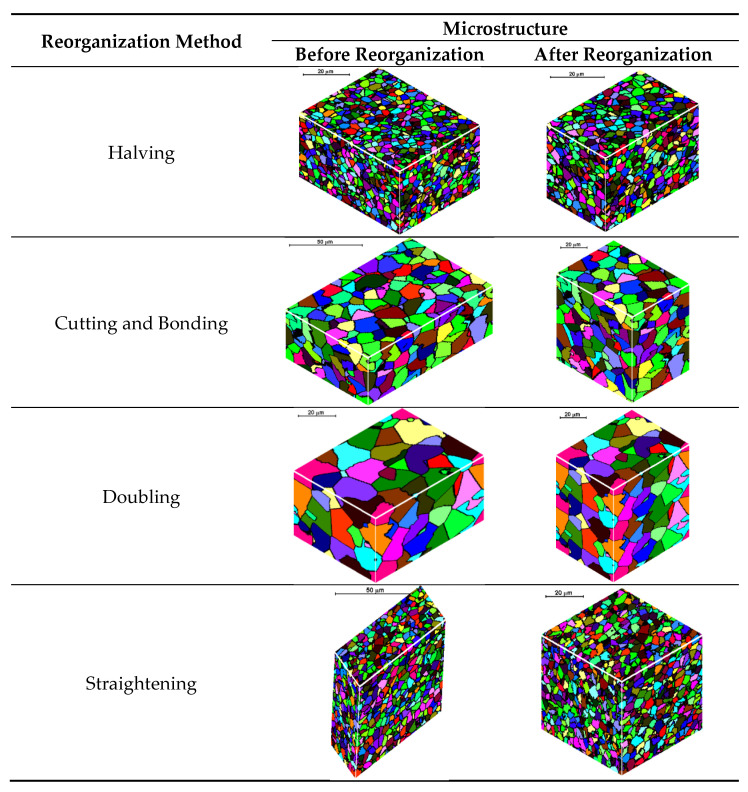
Space reorganization methods.

During the modeling by FCA, it is very important to define the space geometry and boundary conditions as one of the most important parameters. Under certain conditions of deformation, CA space may be distorted, and it may be necessary to reorganize it. It directly affects the boundary conditions, which should be changed. Calculations are optimal with cells of cubic shape, but then, the shape of the cells can be deformed. If the strain accumulated in the material is not large enough, the CA cells change shape and size. When the deformation reaches a predetermined level, the space of the CA should be reorganized to obtain the cubic shape of the cells. Reorganization in CA is similar to “remeshing”, widely used in FEM codes. Different space reorganization methods have been developed (Figure 5). A detailed description of the solutions and algorithms developed in this area can be found elsewhere [26].

### 4.2. Initial Microstructure

**Figure 6:**
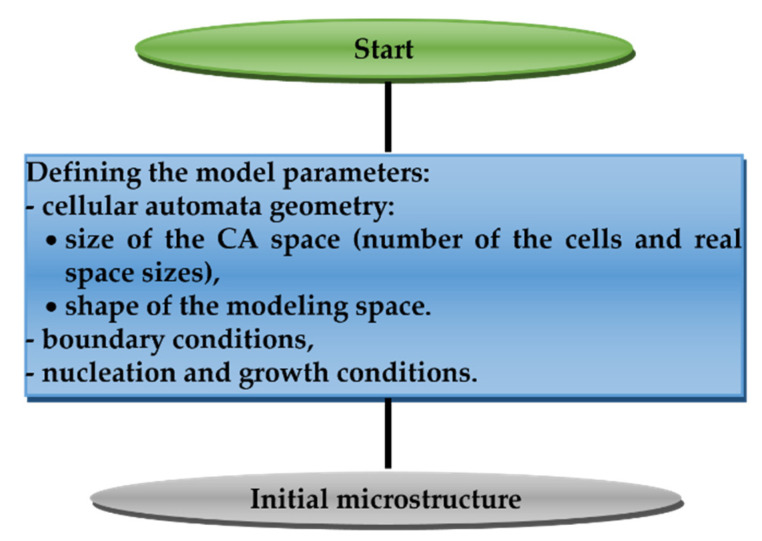
The modeling scheme of the initial microstructure.

Considering the modeling of microstructure evolution in any process, an initial microstructure is necessary, which under certain modeling conditions will be closest to the initial real structure. The developed model of universal frontal cellular automata makes it possible to obtain the initial microstructure, which is the result of two processes or stages: nucleation of grains and their growth. Figure 6 shows the general scheme for modeling the initial microstructure.

The first step in modeling the initial microstructure, based on the three-dimensional model of cellular automata, is the determination of the geometry of the cellular automata by defining the sizes of the three-dimensional space used. This parameter is directly related to the definition of the number of cells for each axis of the system and the actual sizes. An additional factor that is also considered is the shape of the FCA space. The developed model allows using the cuboid or cylinder space. The next step in the process of creating the initial microstructure is to apply the boundary conditions, which may be different in different directions. Information on nucleation is collected and stored by specifying the time and place of appearance of each nuclei and its spatial orientation. This information is ordered according to the time of nucleation. The orientation of the growing grains is also an element that should be considered during the process of creating the initial microstructure. The model considers two types of orientation, i.e., crystallographic orientation and spatial orientation of grain other than spherical. These orientations are combined when modeling the initial microstructure. The model allows for both random selections of the orientation for each grain and allocation of the same orientation for all grains, whose orientation can be chosen randomly or strictly defined. Taking into account all of the factors described above, the model allows modeling of the initial microstructure, which may be different by considering different modeling conditions.

**Figure 7:**
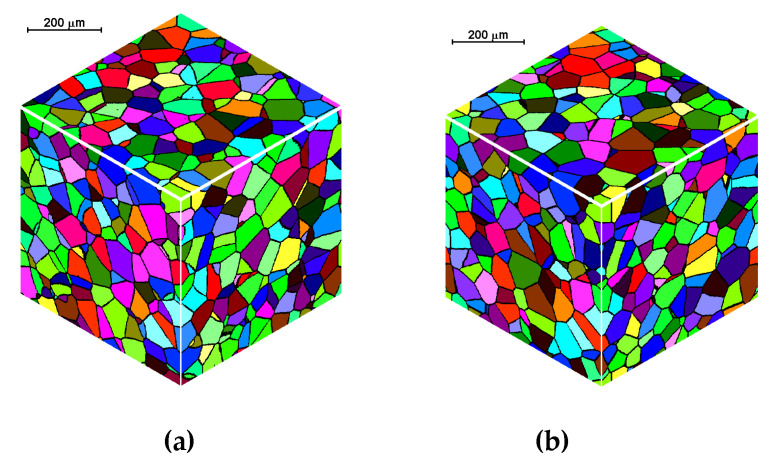
Initial microstructure under different boundary conditions: (**a**) open and (**b**) periodic.

Figure 7 presents the results of the initial microstructure simulation for the spherical shape of the growing grains and open and periodic boundary conditions, with the assumed space of 400 × 400 × 400 cells, the dimensions of the space 500 × 500 × 500 μm, the number of nuclei equal to 600; in each simulation, the nuclei were distributed in the same places in the cell space.

**Figure 8:**
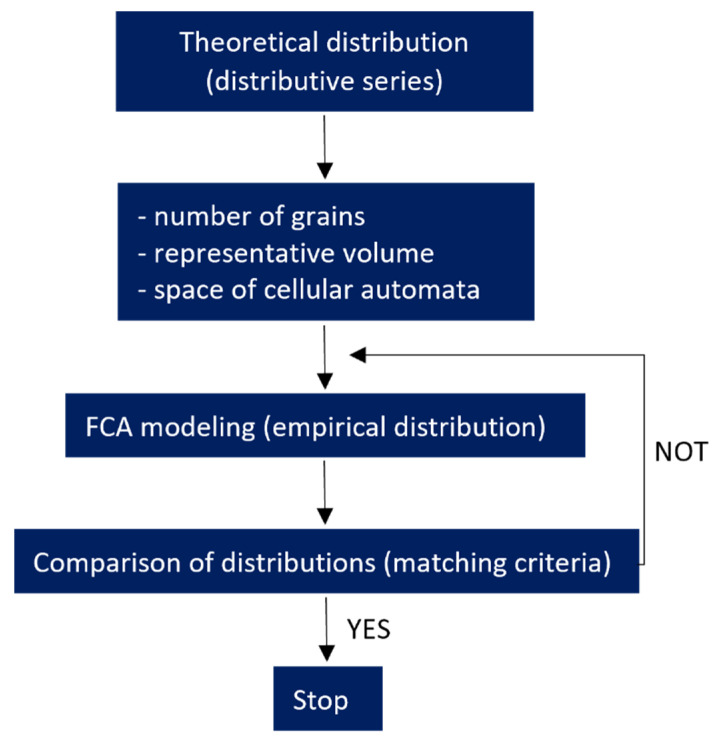
The algorithm for modeling the initial microstructure with a given grain size distribution.

The selection of the boundary conditions and the shape of the growing grains is a preliminary stage preparatory to obtaining the initial microstructure for the given parameters. Such a microstructure, apart from the grain shape, should also meet other requirements concerning, e.g., average grain size, grain size distribution, texture, and others. Obtaining the required grain size distribution in the microstructure can be done in two ways: either by correcting the existing microstructure or by modeling a new microstructure with a matching of the nucleation rate and grain growth. Figure 8 shows schematically the algorithm that allows to obtain a microstructure with a given distribution.

The steps of the algorithm for creating a microstructure with a given grain size distribution are as follows:

1. Representation of the theoretical particle size distribution using a distributive series;
2. Determining the number of grains in a representative model volume and checking the size of the cell space;
3. Defining the nucleation conditions;
4. Modeling the microstructure and determining its empirical distribution;
5. Determining the error of fit by comparing the obtained empirical distribution with the theoretical one;
6. Return to point 3 and perform correction of nucleation conditions in case of failure to meet the matching criterion.

The fulfillment of the matching condition becomes tantamount to obtaining a digital representation of the microstructure with a given grain size distribution and determination of the nucleation conditions. In the case of correction of the existing microstructure, only the first three steps of the algorithm are carried out.

The description of the algorithm that allows obtaining a microstructure with given parameters and examples of simulation results can be found elsewhere [27,28].

### 4.3. Recrystallization

An important factor that influences the microstructure and properties of the material, without which it is impossible to comprehensively consider the processes that take place in many materials, is the phenomenon of recrystallization. In hot-deformed materials, one of the dominant microstructure-reconstruction processes is static recrystallization.

The developed FCA-based model for recrystallization contains several submodels: dislocation density and flow stress model, nucleation model, and grain growth model.

The main assumptions of the recrystallization model are the following:

1. The dislocation density is assigned to the grain, and the initial dislocation density is the same for all grains and equal to a minimal value for current thermal conditions.
2. Nucleation begins when the dislocation density reaches a critical value *ρ_c_*.
3. The nucleation rate depends on the dislocation density, strain, strain rate, and temperature.
4. The migration rate of the grain boundaries is a function of temperature, dislocation density, crystallographic orientation, and other parameters.

Table 1 shows the basic relationships used in the submodels of the recrystallization model. A detailed description of the recrystallization model developed can be found elsewhere [29,30].

**Figure 9:**
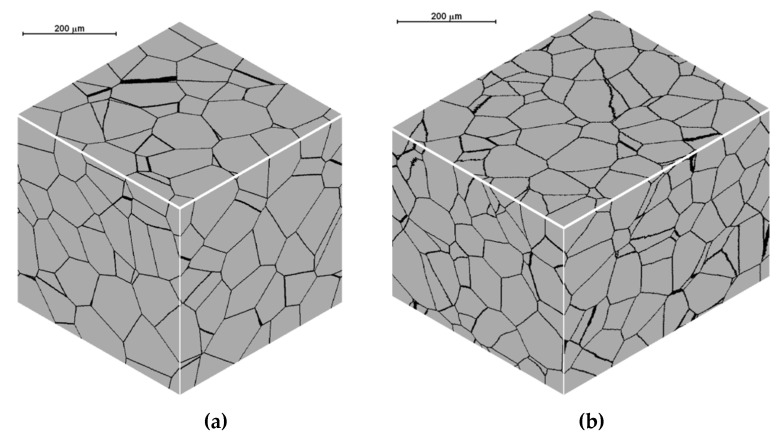
Initial microstructure with an average grain size *d_av_* = 100 µm (**a**) and final microstructure *d_av_* = 75 μm (**b**).

Figure 9 shows the initial microstructure and an example of the final microstructure after deformation at *T* = 1100 °C and complete static recrystallization. The microstructure was obtained for the following conditions: space sizes of 500 × 500 × 500 cells, the dimensions of the space 400 × 400 × 400 μm, and strain rate and strain equal to 1 s^−1^ and 0.18, respectively. The final average grain size was *d_av_* = 75 μm (measured 78.5 μm). The calculations were made for C 45 steel. 

**Figure 10:**
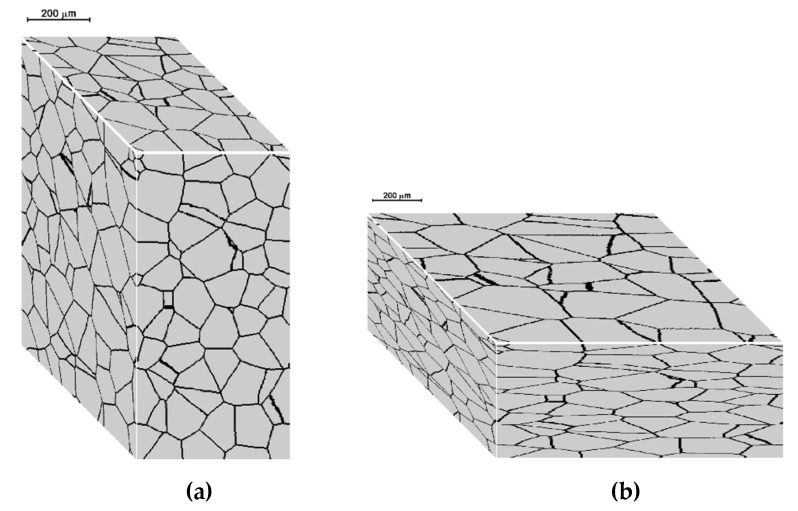

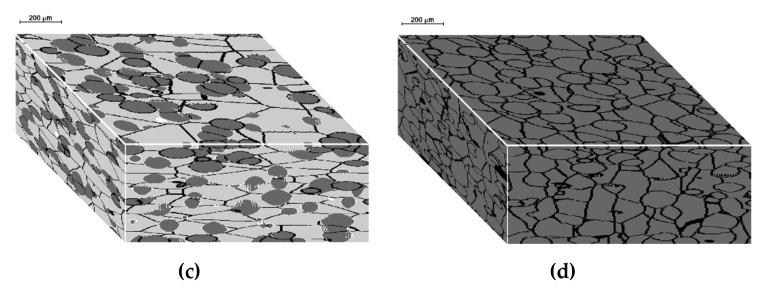
Microstructure before deformation (**a**) and after deformation: without recrystallization (**b**), with dynamic recrystallization (**c**), and after metadynamic recrystallization (**d**).

Figure 10 shows the results of the dynamic and metadynamic recrystallization modeling considering the geometry of the deformation. The initial microstructure before deformation (Figure 10a) and after deformation without recrystallization (Figure 10b) with partial dynamic recrystallization (Figure 10c) and after metadynamic recrystallization (Figure 10d) is shown. Cellular automata with dimensions *n_x_* × *n_y_* × *n_z_* = 200 × 300 × 400 cells and space dimensions *a_x_* × *a_y_* × *a_z_* = 400 × 600 × 800 µm were used for the simulation. The number of grains was 250. The deformation temperature *T* = 1000 °C, the deformation time *t* = 0.5 s, and the nonzero components of the strain rate tensor: ${\dot{\varepsilon }}_{x}=1.0$, ${\dot{\varepsilon }}_{z}=-1.0$, and ${\dot{\varepsilon }}_{xz}=0.07$. The shape of the space and each cell is not a cube in this case but a cuboid. Figure 10b shows the changes in the shape of the space and microstructure with uniform deformation. Figure 10c shows the microstructure with the dynamic recrystallization process that occurs after deformation in 0.5 s. Figure 10d shows the microstructure after full metadynamic recrystallization. The model assumes that nucleation occurs only during deformation, and Figure 10d shows the microstructure after metadynamic recrystallization as an effect of grain growth after dynamic recrystallization.

### 4.4. Phase Transformation

**Figure 11:**
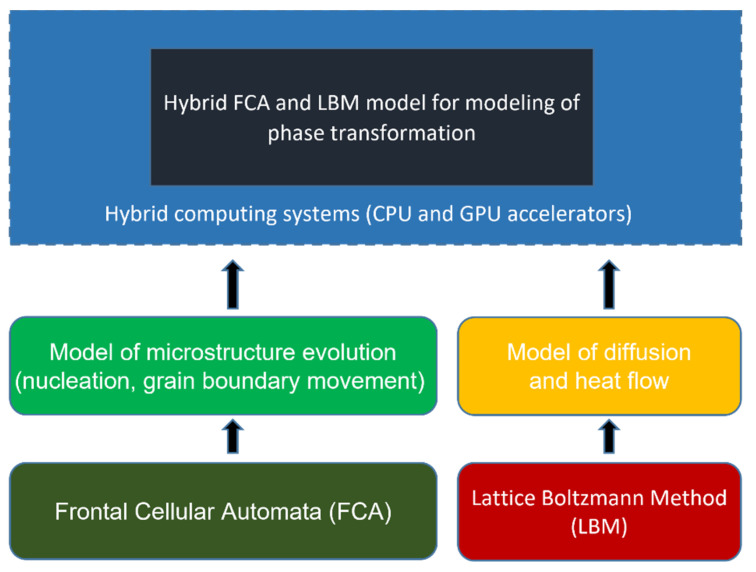
Structure of the hybrid model for modeling diffusional phase transformations.

The main tools for the modeling of phase transformations are cellular automata, often combined with the finite difference method (CA+FDM), phase-field (PF), or multi-phase-field method (MPF). The structure of the hybrid model of diffusion phase transformations developed in carbon steels is shown in Figure 11. The model is based on two modeling methods: FCA and LBM. The model is developed to simulate the evolution of microstructures (FCA), carbon diffusion, and heat flow (LBM).

The model was developed in several stages, from the 1D variant to the 3D variant. At the final stage of model development, the 3D Fourier equation in the following form was used in the calculations:

(19) $$\frac{1}{\alpha }\frac{\partial T}{\partial t}=\left(\frac{\partial^{2}T}{\partial x^{2}}+\frac{\partial^{2}T}{\partial y^{2}}+\frac{\partial^{2}T}{\partial z^{2}}\right)+Q\left(x,y,z,t\right);\frac{\partial c}{\partial t}=D\left(\frac{\partial^{2}c}{\partial x^{2}}+\frac{\partial^{2}c}{\partial y^{2}}+\frac{\partial^{2}c}{\partial z^{2}}\right)$$

where *T*—temperature; *c*—carbon concentration; *α*—thermal diffusivity; *Q*(*x*,*y*,*z*,*t*)—the source of thermal energy; *D*—diffusion coefficient.

The BGK (Bhatnagar–Gross–Krook) model was used for simulations:

(20) $$f_{k}\left(x+\Delta x,y+\Delta y,z+\Delta z,t+\Delta t\right)-f_{k}\left(x,y,z,t\right)=\;-\frac{\Delta t}{\tau }\left[f_{k}\left(x,y,z,t\right)-f_{k}^{eq}\left(x,y,z,t\right)\right]+\Delta tw_{k}S$$

(21) $$f_{k}\left(x+\Delta x,y+\Delta y,z+\Delta z,\;t+\Delta t\right)-f_{k}\left(x,y,z,t\right)\;=-\frac{\Delta t}{\tau }\left[f_{k}\left(x,y,z,t\right)-f_{k}^{eq}\left(x,y,z,t\right)\right]$$

where $f_{k}(x,y,z,t)$, $f_{k}^{eq}(x,y,z,t)$—the particle and equilibrium distribution functions; *τ*—the single-relaxation-time parameter; *S*—the source term; *w_k_*—weighting factor in the direction *k*.

The equilibrium distribution function $f_{k}^{eq}$:

(22) $$f_{k}^{eq}=w_{k}\varphi \left(x,y,z,t\right)$$

where *ϕ*—the dependent variable (temperature *T,* concentration *c*).

Two steps can be considered in LBM calculations:

Collision:

(23) $$f_{i}\left(x,y,z,t+\Delta t\right)=f_{k}\left(x,y,z,t\right)\left[1-\omega \right]+\omega f_{k}^{eq}\left(x,y,z,t\right)$$

where *ω* = Δ*t*/*τ*.

Streaming:

(24) $$f_{i}\left(x+\Delta x,y+\Delta y,z+\Delta z,t+\Delta t\right)=f_{k}\left(x,y,z,t+\Delta t\right)$$

Table 2 shows the developed numerical algorithms that are used in the 3D heat flow and diffusion models. (Steps 2–7 are repeated cyclically).

**Figure 12:**
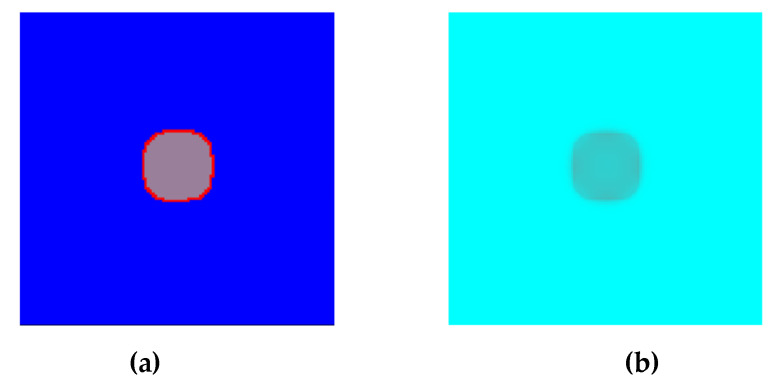
Growth of the new phase in the selected plane: (**a**) CA states and (**b**) temperature distribution.

Figure 12 presents the examples of modeling results for the selected values of the parameters defined in the heat flow algorithm developed: *k_v_* and *k_q_* values. As can be seen, these parameters have a direct impact on the rate of transformation. The *k_v_* is used directly for boundary velocity calculations, while *k_q_* determines the rate of temperature increase in the interface and has an impact on the boundary velocity. The growth of the grain placed in the center of the grid was modelled with the use of the Moore neighborhood and the vector normal to the surface. The calculations were performed on the NVIDIA GeForce GTX 1060 graphics card with the use of CUDA parallel programming. D3Q19 LBM scheme was used for the calculations. The main simulation parameters are as follows: *T_x,y,z_* = 750 °C, *T_P_* = 800 °C, *n_x_* × *n_y_* × *n_z_* = 128 × 128 × 128, *steps* = 100, Δ*x* = 1, Δ*y* = 1, Δ*z* = 1, Δ*t* = 1, *τ* = 1, *k_v_* = 0.003, *k_q_* = 15, and the bounce-back boundary conditions.

The concept of the model and the first 1D modeling results of carbon diffusion and heat flow can be found elsewhere [31,32]. The results of 2D modeling can be found in subsequent work carried out in this area [33].

### 4.5. Modeling of Technological Processes—Rolling Processes

**Figure 13:**
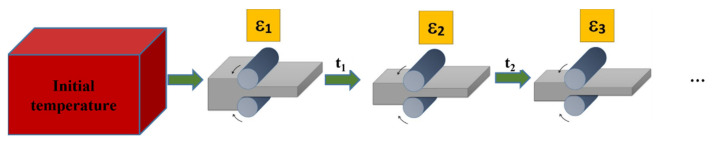
Flat rolling scheme.

The search for modern technological solutions for the rolling process, which refers to the acquisition of higher-quality rolled products and reducing the cost of their production, becomes a necessity in the modern stage of development of this technological process. Often, during rolling, it is difficult to simultaneously ensure product dimensions and shape and the required microstructure, which is responsible for the final mechanical properties. The intensive development of computer technology makes it easier to use modern numerical methods for the simulation of complex forming processes. The developed modeling system can be applied effectively to model microstructure evolution in the rolling processes of various materials. General characteristics of flat rolling is required for the modeling of the process (Figure 13). Analytical solutions and advanced numerical models can be used to obtain the process parameters and their average values. FCA modeling is based on the information about the duration of plastic deformation, the time intervals between the deformations, temperature, strain rate, etc.

The developed modeling system can be adapted to model various materials. The overall model of this process has been comprehensively verified, for example, for modeling the evolution of the microstructure in AISI 304L stainless steel [34].

**Figure 14:**
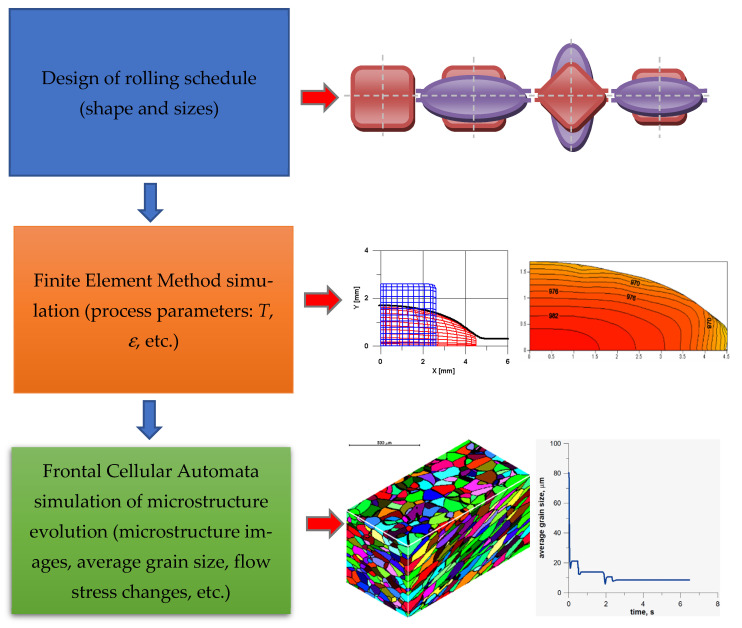
The general scheme of shape rolling modeling.

Simulations of microstructure evolution can be a part of more complex modeling system or performed independently. It can be realized in three modeling stages (Figure 14): design of rolling schedule, finite element method simulation, and frontal cellular automata simulation of microstructure evolution. The second and third stages can be repeated several times.

A detailed description of first stage and the designed schedule can be found elsewhere [35]. The examples of simulation results of the second stage can be found in detail elsewhere [36]. The third stage includes the FCA simulation of the microstructure evolution, which can be performed for any representative point (for which data can be obtained) using any FEM code. For the calculation, information on the time, temperature, and strain rate tensor are used. More information on this stage can be found elsewhere [37]. The developed shape rolling scheme can be applied, in principle, to any material. Simulation, prediction, and validation of the evolution of microstructures in AISI 304L stainless steel during the shape rolling process were presented in one of the unpublished works [38]. Other results can be found elsewhere [39].

### 4.6. Grain Refinement and Modeling of Severe Plastic Deformation

The main principles of severe plastic deformation (SPD) modeling based on grain refinement using the presented FCA platform are described in [40] and their references. Then, several SPDs were modeled: accumulative roll bonding process and MaxStrain technology [41,42]. 

Another innovative combined metal forming process, which can be treated as one of the SPD techniques, consists of three different modes of deformation: asymmetric drawing with bending, namely accumulated angular drawing, wire drawing, and wire flattening, which was also modeled on the FCA platform [43].

The evolution around highly reactive interfaces in the processing of nanocrystallized multilayered metallic materials has been investigated and discussed in [44].

## 5. LBM-Based Platform for Modeling of Advanced Additive Manufacturing Characterized by the Changes of State of Matter

Authors took part in a development of a new modeling platform presented firstly in [45]. Additive manufacturing (AM) technologies that contain phase transitions are a main subject for the three-dimensional simulation on the platform. It is based on LBM with CA elements and was primarily oriented on selective laser melting (SLM). An example of calculation of the model parameters for the real material (Ti-6Al-4V alloy) and the real process is presented in [45]. It contains also the first quantitative results. It can be used for analysis of new multipasses and multimaterials SLM processes and can be served for computer-aided design. The principles and evolution of the development platform can be found by tracking previous publications presented in the references of the cited publications [45]. Other aspects of this LBM-CA platform can be found elsewhere [46,47]. Some essential details of the platform are presented below.

**Figure 15:**
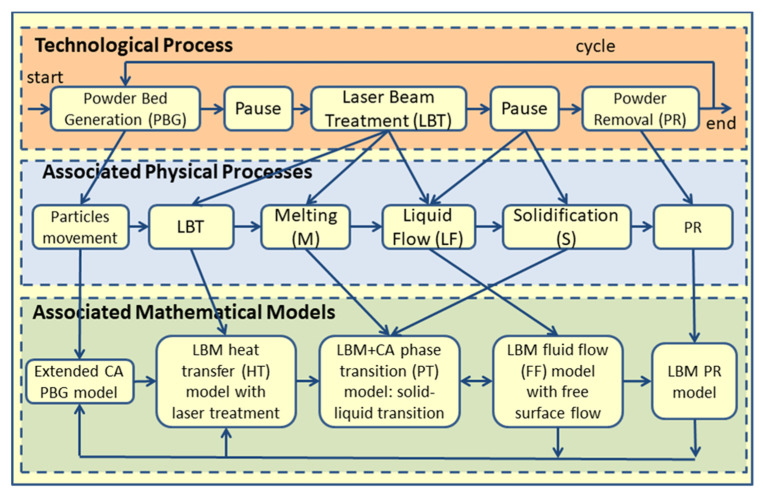
Schematic representation of interconnection between analyzed process physical phenomena and models [45].

Figure 15 represents the proposed scheme, where the process is divided into different stages according to the associated physical phenomena, which are related to the corresponding mathematical models.

The powder bed generation (PBG) model with some results was published in [46,48]. The first a simplified model of heat transfer was presented in [48,49]. The CA method was the basis of these two models. For the other models, the LBM was used [45,47,49]. 

In this paper, we present a model of powder particles removal that was developed on the general principles of the platform with the use of LBM, as in Section 3. It can be also used for optimization of micro vacuum material removal system. The main LBM blocks for gas flow in this model are the same (Figure 3). The same velocity model (D2Q9) is applied. The model contains equations that describe the particles motion in the gas. The new horizontal *v_x_* and vertical *v_y_* velocity components are calculated as follows:

(25) $$v_{x}^{p}\left(t+1\right)=mv_{x}^{p}\left(t\right)+\left(1-m\right)v_{x}\left(t\right)$$

(26) $$v_{y}^{p}\left(t+1\right)=mv_{y}^{p}\left(t\right)+\left(1-m\right)v_{x}\left(t\right)-g$$

where *m*—particle mass; *g*—gravity. The mass *m* and gravity *g* are dimensionless in the models. The mass *m* varies from zero ( *m* = 0, very small, light particles) to unity (*m* = 1, a very heavy particle). The gravity *g* depends on the modeling parameters: lattice size, time step, and particle size. For very small particles, when the gas resistance is high, *g* = 0. For big, very heavy particles, the lattice size and time step define the gravity, *g* = *g*_max_.

The location of a particle is a continuous variable, while the velocity field is discretized. A new position is calculated according to simple Euler’s integration scheme:

(27) $$x^{p}\left(t+1\right)=x^{p}\left(t\right)+\frac{u_{x}^{p}\left(t+1\right)+u_{x}^{p}\left(t\right)}{2}$$

(28) $$y^{p}\left(t+1\right)=y^{p}\left(t\right)+\frac{u_{y}^{p}\left(t+1\right)+u_{y}^{p}\left(t\right)}{2}$$

**Figure 16:**
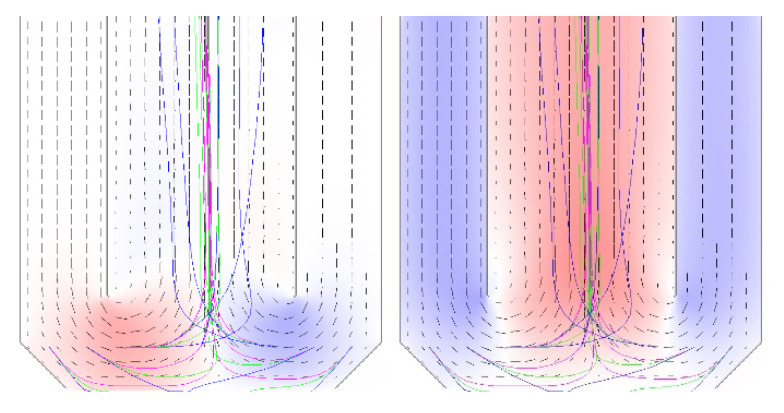
Modeling results showing inflows and outflows in the removal equipment.

An analysis of simulation results shows that particle removal with the use of only a vacuum function is highly dependent on the gap. When the gap is wider, the effectiveness is lesser, and the control of the gap is difficult. To enforce movement in the opposite direction and turbulences, an additional inflow channel with much higher gas pressure was proposed. An example of a simulation result is presented in Figure 16. The modelling space was 128 × 128 cells or nodes. To make this scheme more effective, strong gas stream is forced in the horizontal direction. Other designs of removal systems can be analyzed by this model.

**Figure 17:**
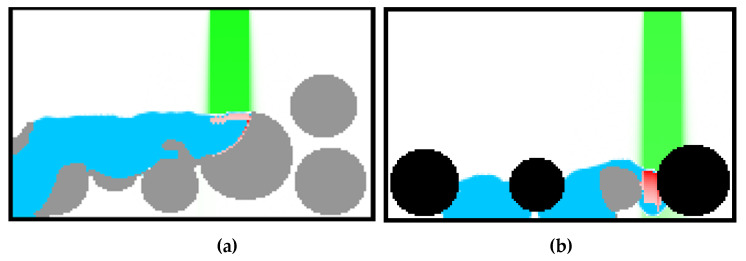
Simulation results of the SLM process: (**a**) one material and (**b**) two different materials.

In Figure 17, examples of two cases of SLM simulation are presented. The modeling space was 128 × 80 nodes. The presented results were obtained with the use of sequential FORTRAN code on PC with Intel Core i7-3930 K, and calculations lasted about 3 h. The similar simulations with parallel computations on GPU with CUDA technology using the graphic card GeForce 1060 with 1280 CUDA cores lasted about 1.5 min, that is, about 100 times faster. The solid material is presented in gray and black, while the liquid material is blue. The laser beam is shown by the green color. The intensity of heat transfer from the laser beam to the materials is correspondent to intensity of pink and red colors. The case of one material is presented in Figure 17a, whereas the case of two different materials is presented in Figure 17b. It can be seen that the processing of two materials with a significant difference in material properties is accompanied by the problem of melting the material with a higher melting temperature, and solving this problem is one of the main goals of here-presented development platform.

**Figure 18:**
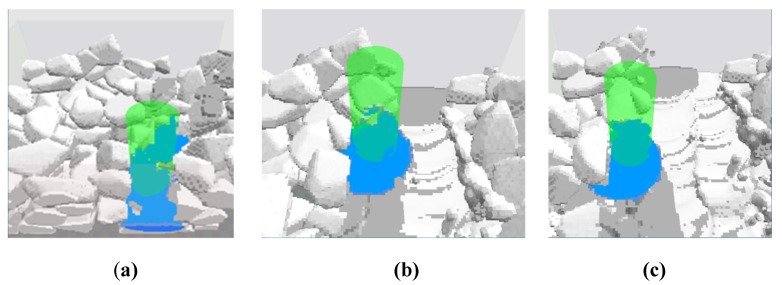
3D perspective views of the modeled SLM process with three passes: (**a**) the first, (**b**) the second, and (**c**) the third.

An example of modeling a 3D selective laser melting is presented in Figure 18. The following conditions were assumed for the calculations: the average particle size is about 35–40 μm, the model space is *x* × *y* × *z* = 256 × 128 × 64 cells, the laser travel velocity is 1 m/s, and the laser power was equal to 200 W. Three passes are presented. The gray color represents particles of powder and solidified material. The blue color corresponds to the liquid phase of the material. The laser beam is shown in green. Other examples can be found elsewhere [45].

## 6. Conclusions

The paper presents basic information about modeling platforms based on relatively new and little-known methods for modeling phenomena and processes in materials science and engineering FCA and LBM.

The first platform for modeling microstructure evolution was first based on FCA, which is very suitable for modeling processes of multiple nucleations and grain growth in materials and allows for algorithmic acceleration of calculations with maintaining high accuracy. Then, this platform was supplemented by the LBM, which is widely used not only in hydrodynamics but also for modeling heat flow or diffusion processes. LBM can also become the basic method for modeling complex processes and phenomena containing not only flows but also different transformations of both the first and second type: both state and phase transformations, including flow, diffusion, heat transfer, latent heat, and many other phenomena that are difficult to consider with the use of other methods. The components of the systems and their role in the comprehensive modeling of various processes are shown. The first platform allows modeling of recrystallization, crystallization, and phase transformation, and it is mainly oriented to model forming processes (flat, shape rolling, drawing, SPD, etc.). The performance of the systems was verified in application to specific materials and process parameters, and the selected modeling results are presented.

The large variety of modeling phenomena creates opportunities for the expansion of complex models that are homogeneous and do not need multiscale modeling with a complex slow-acting interface between different methods. This aspect eliminates the possibility of efficient and fast modeling. An advantage of using FCA and LBM is the ease of parallelization of calculations on modern GPUs using, for example, CUDA software. The use of several or tens of thousands of GPU processors allows accelerating the calculations several hundred times. The developed systems also take into account solutions based on parallel calculations, and examples of the results are shown.

The second platform for the modeling, design, and optimization of additive manufacturing is in development and is based on LBM and CA. Elimination of complicated interfaces allows for modeling of the manufacturing process within the single integrated platform. The platform is ready to be used for computer-aided analysis, optimization, and design of multimaterial, multipass SLM cycles.

The second platform can be adapted for several other additive manufacturing (AM) technologies that contain phase transitions (melting-solidification). Mainly, they are included in the group of powder bed fusion techniques (PBF): DMLS (direct metal laser sintering), SLS (selective laser sintering), MJF (multi-jet fusion), and EBM (electron beam melting). It can be used for modeling other AM, for example, WAAM (wire arc additive manufacturing) and FFF (fused filament fabrication), also known as FDM (fused deposition modeling), etc. However, it requires additional efforts for the adaptation of existing models and the development of missed ones.

The second platform can be expanded with modeling by CA (FCA) of microstructure formation and evolution during the solidification.

## Figures and Tables

**Table 1:** Recrystallization model.

Submodels	Dependencies	
Dislocation Density and Flow Stress Model	$\sigma =\sigma_{0}+\alpha \mu b\sqrt{\rho_{av}}$	(10)
	$\dot{\rho }=U\left(\varepsilon \right)\dot{\varepsilon }-\Omega \left(\rho \right)\dot{\varepsilon }-R\left(\rho \right)$	(11)
Nucleation Model	${\dot{n}}_{V}=a_{N}\varepsilon^{l_{N}-1}D_{0}^{-k_{N}}\dot{\varepsilon }$	(12)
	$\Delta {\dot{n}}_{V}=a_{N}\varepsilon^{l_{N}-1}D_{0}^{-k_{N}}\dot{\varepsilon }\left(N_{Vmd}-N_{V}\right)\Delta t$	(13)
	$N_{Vmd}=a_{md}{\dot{\varepsilon }}^{-m}exp{\left(\frac{Q}{RT}\right)}^{-m}$	(14)
	$D_{srx}=A\varepsilon^{a}D_{0}^{b}Z^{c}$	(15)
	$Z=\dot{\varepsilon }exp\left(\frac{Q}{RT}\right)$	(16)
Grain Growth Model	v=Mf(θ)F	(17)
	$\{\begin{array}{ccc} f=0 & dla & \theta <7^{{}^{\circ}}\\ f=3.72*10^{-4}exp\left\{8*{\left[1-exp\left(-\frac{\theta }{2.2}\right)\right]}^{10}\right\} & dla & 7^{{}^{\circ}}<\theta <15^{{}^{\circ}}\\ f=1 & dla & \theta >15^{{}^{\circ}} \end{array}$	(18)

**Table 2:** Algorithms for 3D simulation of heat flow and carbon diffusion.

Heat Flow	Diffusion
Initial parameters (1)	
Number of nodes: *n_x_, n_y_*, and *n_z_*, node position: *x*, *y*, *z*, number of time steps: *tsteps*, grid step: Δ*x*, Δ*y*, Δz, time step: Δ*t*, austenite and ferrite fractions in interface node: *φ_IA_* = 1, *φ_IF_* = 0 (1a)	
Velocity and specific enthalpy coefficients: *k_v_* and *k_q_*, temperatures of phase transformation, in the interface, and in the node: *T_P_*, *T_I_*, and *T_x,y,z_* (1b)	Carbon concentration in nodes: *c_i_*, diffusion coefficient: *D_cf_* (1b)
Calculations of a boundary velocity, the quantity, mass, or volume of the transformed material in the interface node; fraction of the new phase in the interface node; fraction checking (2)	
*v* = *k_v_*(*T_p_* − *T_I_*) (2a)	*v* = Δ*c*; (2a)
Δ*φ_I_* = *v*Δ*t*; *φ_IF,t_* = *φ_IF,t−1_* + Δ*φ_I_* (2b)	
If *φ_IF,t_* < 1 ⇒ go to step (2e) (heat source) (2c)	if *φ_IF,t_* < 1 ⇒ go to step (3) (*c* calculation) (2c)
If *φ_IF,t_* ≥ 1 then: *ϕ_IF,t_*’ = 1, change the state of node from interface to ferrite and the neighboring nodes from austenite to interface (according to neighborhood), set the value of the fraction for the new interface: Δ*φ_nI_* = (*φ_IF,t_* − *φ_IF,t_*’)/*numA* = *φ_IF,nI_*; *numA*—the number of nodes in phase austenite in the neighborhood of old interface, the Δ*φ* for the old interface (new ferrite) node: Δ*φ_oI_* = 1 − φ*_IF_*_,*t*−1_; *φ_IF,oI_* = 1; *φ_IA,oI_* = 0 (2d)	
Heat source calculation: if *φ_t_* < 1 ⇒ *Q_I_* = *k_q_*Δ*φ_I_* if *φ_t_* ≥ 1 ⇒ *Q_nI_* = *k_q_*Δ*φ_nI_; Q_oI_* = *k_q_*Δ*φ_oI_* (2e)	
Determination of new temperature: *T_x,y,z_* = *Σf_i_* + *Q_x,y,z_* (3)	Determination of new concentration: *c* = Σ*f_i_* (3)
*f^eq^* calculations (4)	
Collision (5)	
Streaming (6)	
Boundary conditions (7)	

## Data Availability

Not applicable.
